# Mutation of the GDP-Fucose Biosynthesis Gene *gmds* Increases Hair Cell Number and Neuromast Regenerative Capacity in Zebrafish

**DOI:** 10.3390/ijms26199737

**Published:** 2025-10-07

**Authors:** Muhammad T. Ameen, Gerissa Fowler, Curtis R. French

**Affiliations:** Division of Biomedical Sciences, Faculty of Medicine, Memorial University of Newfoundland, St. John’s, NL A1B 3V6, Canada; mtameen@mun.ca (M.T.A.); gerissa.fowler@oncology.ox.ac.uk (G.F.)

**Keywords:** zebrafish, fucosylation, *GMDS*, neuromast, hair cell, regeneration, hearing loss

## Abstract

Hearing loss affects millions and is often caused by irreversible damage to mechanosensory hair cells. Humans and other mammals lack the capacity to regenerate damaged hair cells; however zebrafish, *Danio rerio*, can regenerate hair cells that are present in the ear and mechanosensory neuromasts, making this animal an ideal model for understanding hair cell regenerative mechanisms. This study investigates the role of the GDP-fucose biosynthesis gene *GDP-mannose 4,6-dehydratase* (*gmds*) in regulating neuromast hair cell regeneration in zebrafish. Fucosylation is required for Notch signalling, a critical negative regulator of hair cell regeneration, and we therefore hypothesized that loss of *gmds* function would enhance hair cell regeneration. We demonstrate increased hair cell number in *gmds* mutants, and increased hair cell number following chemical ablation of hair cells with neomycin. Additionally, *gmds* mutants exhibited accelerated neuromast and hair cell regeneration, achieving complete restoration faster than wild-type siblings. Pharmacological inhibition of Notch signalling further enhanced hair cell regeneration in wild-type siblings but less so in *gmds* mutants, indicating that Notch signalling may partially regulate hair cell regeneration downstream of *gmds*. These findings highlight the importance of GDP-fucose biosynthesis in regulating hair cell number and regeneration, likely partially dependent on Notch signalling.

## 1. Introduction

Approximately 430 million people suffer from hearing loss [1], the majority of which result from damage to the mechanosensory hair cells in the inner ear. Damage to hair cells is caused by normal ageing, noise pollution or intake of ototoxic drugs such as aminoglycosides for treating bacterial infection or cisplatin used in cancer treatment [2]. Current treatments for hearing loss include hearing aids and cochlear implants which partially restore hearing but do not address the underlying cause of hair cell damage. Inner ear hair cells in humans are specialized sensory cells required for detecting mechanical stimuli such as sound vibrations and converting them into electrical signals that are processed by the brain. Unlike humans, non-mammalian species such as fish, birds, reptiles and amphibians have the ability to regenerate hair cells after damage [3]. More effort is therefore needed to understand genes and signalling pathways that trigger hair cell regeneration, so that they may be eventually manipulated in humans for hearing loss treatment.

Zebrafish are commonly used to study hair cell regeneration mechanisms due to their ability to regenerate hair cells throughout their lifespan [4,5]. Zebrafish possess hair cells in otic sensory epithelia and in mechanosensory organs called neuromasts. The structure and function of these hair cells are similar to humans and notably, mutations disrupting the function of zebrafish hair cells have also been reported to cause deafness in humans [6,7]. To detect mechanical stimuli created by water motion through mechanotransduction, zebrafish hair cell apical structures called stereocilia (hair bundles) are arranged at different heights close to a kinocilia at the centre. Water motion deflection of the stereocilia towards the kinocilia causes the opening of force-gated mechanoelectrical transduction (MET) channels [8] on the kinocilium, with corresponding electrical stimuli sent to the hindbrain by innervating bipolar neurons [9]. Using zebrafish, putative genes that regulate hair cell function or regeneration can be manipulated with gene editing techniques with the resulting effects on neuromast morphology, function and regeneration visualized using vital dyes and electron microscopy.

Signalling pathways such as Wnt, Notch and Fgf are involved in hair cell regeneration [10,11,12]. Activation of Wnt signalling has been shown to increase the proliferation of the neuromast support cells via their return to the cell cycle to generate new hair cells after damage [13], a process normally inhibited by Notch and Fgf signalling [5,10,14,15,16]. This indicates the need for a transient downregulation of Notch and Fgf signalling for increased Wnt dependent proliferation of the supporting cells immediately after hair cell damage. However, Notch and Fgf signalling are still required in the later stages of regeneration for differentiation, cell fate specification and to limit excessive hair cell production [10,17].

The *gmds* gene encodes GDP-mannose 4,6-dehydratase enzyme required for the de novo synthesis of GDP-fucose in cells. This enzyme catalyzes the rate limiting reaction of GDP-fucose de novo synthesis and accounts for about 90% of GDP-fucose availability in cells, while a salvage pathway that recycles GDP-fucose from glycoproteins and glycolipids contributes around 10% of the available cellular GDP-fucose [18,19]. *gmds* is expressed in neuromast support cells [10,20], and GDP-fucose is essential for fucosylation and signalling activity of Notch receptors [21,22,23,24] and could therefore play a role in hair cell regeneration. Furthermore, *gmds* is downregulated in *fgf3* mutants that have increased hair cell number during homeostasis and increased hair cell regenerative capacity [5,10]. This study aims to investigate the role of *gmds* loss of function on hair cell regeneration using a CRISPR-generated *gmds* mutant zebrafish line [20]. We hypothesized that *gmds* mutant zebrafish may have defects in neuromast hair cell number during homeostasis and will generate neuromast hair cells faster and in abundance after damage with neomycin, potentially via the downregulation of Notch signalling.

## 2. Results

### 2.1. Abnormal Fucosylation but Normal Neuromast Morphology in gmds^−/−^ Mutants

**Figure 1:**
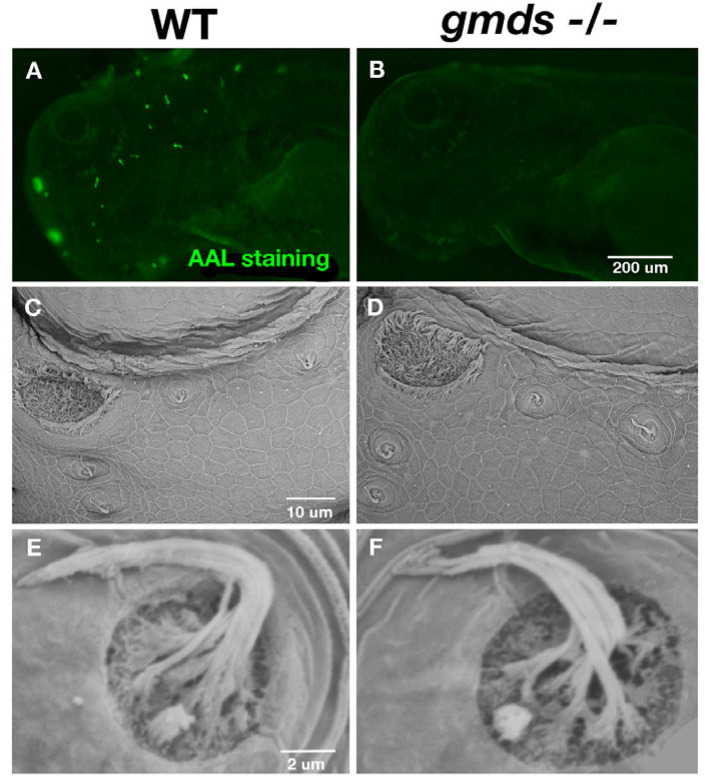
Abnormal fucosylation but normal neuromast morphology in *gmds^−/−^*. *Aleuria aurantia* lectin staining for fucosylated proteins (**A**,**B**) is present on control (*n* = 20), but not *gmds^−/−^* neuromasts (*n* = 14). Scanning electron microscope (SEM) image of the lateral line neuromasts at 5 dpf under homeostatic conditions in wild-type (*n* = 9) and *gmds^−/−^* (*n* = 9) (**C**,**D**) with a higher magnification image of a supraorbital neuromast in (**E**,**F**).

To determine how the loss of *gmds* in homozygous mutants affects the level of fucosylated proteins in zebrafish, we used *Aleuria aurantia* lectin (AAL) staining to assess wild-type and homozygous mutant fucosylation levels at 3 dpf. Figure 1B shows a depletion of fucosylated proteins in *gmds^−/−^* neuromasts compared to wild-type larvae in Figure 1A. We assessed neuromast and hair cell structure in *gmds* embryos using SEM, demonstrating that all neuromasts are present and morphologically similar in wild-type siblings (Figure 1C–F).

### 2.2. Circular Swimming and Increased Hair Cell Number in gmds Mutant Neuromasts Under Homeostatic Conditions

**Figure 2:**
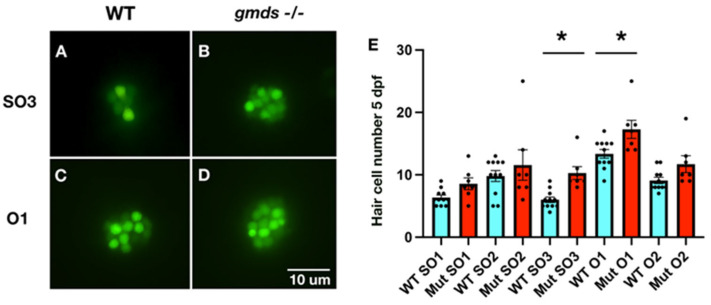
Increased neuromast hair cell number in *gmds^−/−^* mutants under homeostatic conditions. The SO3 and O1 neuromasts show a statistically significant increase in hair cell number at 5 dpf in *gmds^−/−^* mutants when compared to control siblings, as evidenced by YO-PRO-1 staining (**A**–**D**). While the other quantified neuromasts (SO1, SO2, O2) show a trend of increased hair cell number under homeostatic conditions, these data were not statistically significant (**E**). Data from two experiments (WT *n* = 11, Mut *n* = 7). Data presented as mean ± SEM, significance testing using a one-factor ANOVA with Sidak’s multiple comparisons test * *p* < 0.05.

Homozygous *gmds^−/−^* fish swim in circles [Supplemental data, Video S1 (WT) and Video S2 (*gmds^−/−^*)], often regarded as a deafness phenotype that involves neuromast dysfunction [6,25,26,27]. To assess hair cell number in *gmds* mutant embryos, we stained hair cells with YO-PRO-1 at 5 dpf (Figure 2). Quantification of hair cells in five neuromasts (Supraorbital neuromasts SO1, SO2 and SO3) and otic neuromasts (O1 and O2) revealed a trend of increased cell number (Figure 2E). In all five neuromasts, increased numbers of hair cells were quantified with the SO3 and O1 neuromasts having a statistically significant increase under homeostatic conditions (Figure 2A–E). Circular swimming may also be influenced by altered synaptic density at the neuromuscular junctions, as both increased [28] and decreased synaptic density is observed amongst homozygous *gmds* mutants (Supplemental Figure S1).

### 2.3. Neuromast Markers Are Not Altered in gmds Mutants Under Homeostatic Conditions

**Figure 3:**
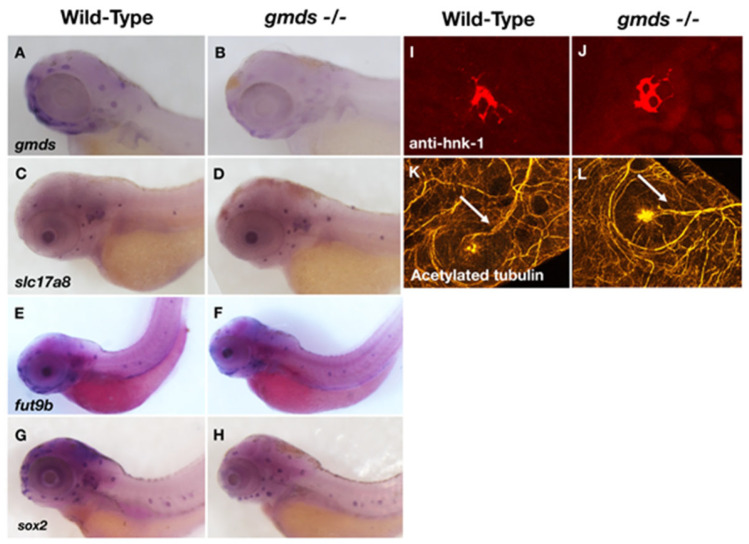
No change in neuromast genetic markers or neural innervation in *gmds^−/−^* mutants. Expression level of the *gmds* gene at 3 dpf shows downregulation in homozygous mutants when compared to control siblings (**A**,**B**). No changes are observed in the expression of *slc17a8* (**C**,**D**), the neuromast-specific fucosyltransferase *fut9b* (**E**,**F**) or the support cell specific *sox2* (**G**,**H**) between *gmds^−/−^* and control siblings at 3 dpf. In situ hybridization experiment was repeated three times for each genetic marker (WT *n* = 15, *gmds^−/−^ n* = 15), with pictures at 40X. Neuromasts are innervated, with both *gmds^−/−^* and control siblings staining positive for Hnk-1 (**I**,**J,** pictures taken at 200X**)** and acetylated tubulin (**K**,**L,** pictures taken at 630X) using immunohistochemistry from two independent experiments (WT *n* = 6, *gmds^−/−^ n* = 6). Arrows point to nerve innervation in K and L.

To check whether the CRISPR *gmds* loss of function mutation in zebrafish causes disruption of genes expressed in neuromast hair cells, in situ hybridization was used at 3 dpf. Depleted expression of *gmds* mRNA in the *gmds^−/−^* mutants, showing a successful loss of function via nonsense-mediated decay, was observed as previously reported [20,29] (Figure 3A,B). No change was observed in the neuromast-specific fucosyltransferase *fut9b* (Figure 3E,F), nor were there any changes in the expression of genes previously implicated in human hearing loss such as *slc17a8* [30,31] and *sox2* [32,33] (Figure 3C,D,G,H). Zebrafish neuromasts are innervated, with positive staining for Hnk-1 and acetylated tubulin in both control and *gmds^−/−^* neuromasts (Figure 3I–L).

### 2.4. Increased Neuromast Regeneration Capacity of gmds^−/−^ Mutants with DASPEI Staining

**Figure 4:**
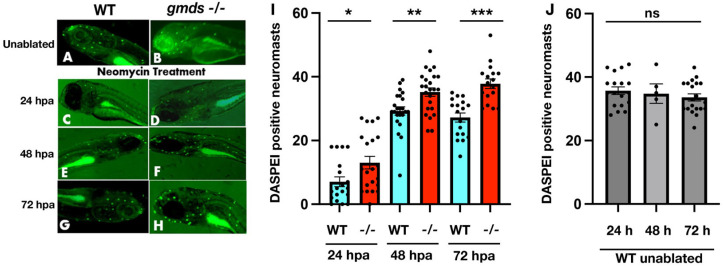
Increased neuromast regeneration capacity of *gmds^−/−^* mutants with DASPEI Staining. WT and *gmds^−/−^* mutant unablated neuromasts at 6 dpf from four independent experiments (WT *n* = 17, *gmds^−/−^ n* = 18) (**A**,**B**). Wild-type and *gmds^−/−^* mutants show a statistically significant difference in the number of labelled neuromasts 24 h post neomycin ablation. For four experiments, WT *n* = 17, *gmds^−/−^ n* = 19 (**C**,**D**,**I**). A further increase is observed at 48 hpa WT *n* = 23, *gmds^−/−^ n* = 24 (**E**,**F**,**I**) and 72 hpa WT *n* = 18, *gmds^−/−^ n* = 17 (**G**–**I**). Control siblings quantified at the same time points show no difference in neuromast number (**J**). Images taken at 40X. Data presented as mean ± SEM, significance testing using a one-factor ANOVA with Sidak’s multiple comparisons test * *p* < 0.05, ** *p* < 0.01, *** *p* < 0.001. ns = not significant.

To test our hypothesis for increased regeneration rates in *gmds^−/−^* lateral line neuromast hair cells after ablation, we first used fluorescent dye 2-[4-(dimethylamino)styryl]-N-ethylpyridinium iodide (DASPEI; Molecular Probes, Eugene, OR, USA) staining to quantify the number of metabolically active neuromasts after ablation with neomycin. A significant difference was observed in the total number of regenerated lateral line neuromasts between WT and *gmds^−/−^* 24 h post-ablation (hpa) with neomycin (Figure 4C,D,I). This difference became more pronounced by 48 and 72 h post-treatment, where the total number of regenerated neuromasts in *gmds^−/^^−^* was again significantly more than the neomycin-treated control embryos (Figure 4E–I). By the 48 h post-ablation time point, *gmds^−/−^* mutants had a similar number DASPEI positive neuromasts to untreated wild-type larvae (Figure 4A,B,I,J).

### 2.5. Scanning Electron Microscopy Imaging of Neuromasts

**Figure 5:**
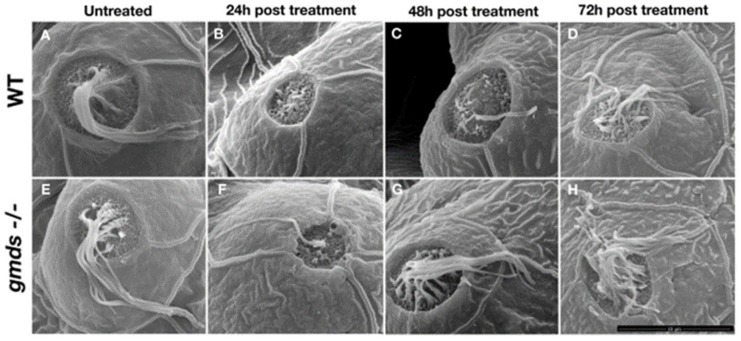
Increased rate of regeneration of larval head neuromasts after neomycin ablation. Neuromasts are morphologically similar in control and *gmds^−/−^* mutants at 5 dpf without neomycin ablation (**A**,**E**). Little difference is seen in neuromast morphology at 24 hpa (**B**,**F**), but by 48 hpa, neuromast morphology is normal in *gmds^−/−^*, implying complete regeneration (**G**), while control siblings have not yet fully regenerated (**C**). By 72 hpa, both control and *gmds^−/−^* neuromasts appear fully regenerated (**D**,**H**). Data shown from three experiments for each condition or time post-treatment, WT *n* = 9, *gmds^−/−^ n* = 9. Scalebar in H is 10 μm.

**Figure 6:**
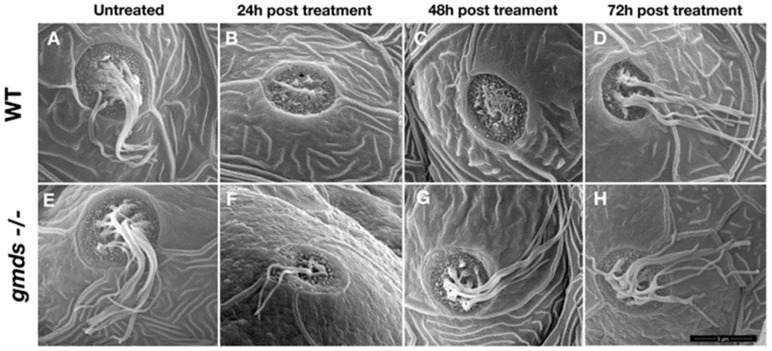
Increased rate of regeneration of larval tail neuromasts after neomycin ablation. Neuromasts are morphologically similar in control and *gmds^−/−^* mutants at 5 dpf without neomycin ablation (**A**,**E**). Little difference is seen in neuromast morphology at 24 hpa (**B**,**F**), but by 48 hpa, neuromast morphology is normal in g*mds^−/−^*, implying complete regeneration (**G**), while control siblings have not yet fully regenerated (**C**). By 72 hpa, both control and *gmds^−/−^* neuromasts appear fully regenerated (**D**,**H**). Data shown from three experiments for each condition or time post-treatment, WT *n* = 9, *gmds^−/−^ n* = 9. Scalebar in H is 5 μm.

To confirm increased rate of regeneration in *gmds* mutants and to examine the structural integrity of the regenerated lateral line neuromast and hair cells, we performed scanning electron microscopy on regenerated neuromasts. Again, no morphological difference was observed in the zebrafish head neuromasts in untreated embryos (Figure 5A,E), with similar results in the trunk neuromasts (Figure 6A,E). 24 h post neomycin treatment, both the wild-type and *gmds^−/^^−^* have had their neuromast hair cells ablated (Figure 5B,F, head neuromasts, Figure 6B,F, trunk neuromasts), with the vast majority of kinocilia and stereocilia absent. However, at 48 h post-treatment, almost all the hair cells of the *gmds^−/−^* neuromast were regenerated with kinocilia and stereocilia present, while control (wild-type or heterozygotes) larvae were not fully regenerated (Figure 5C,G and Figure 6C,G). Hair cell regeneration appears complete in both control and *gmds^−/−^* by 72 h post neomycin ablation (Figure 5D,H and Figure 6D,H).

### 2.6. Quantitative Assessment of Increased Hair Cell Regeneration Capacity of gmds^−/−^ Mutants with YO-PRO-1 Staining

**Figure 7:**
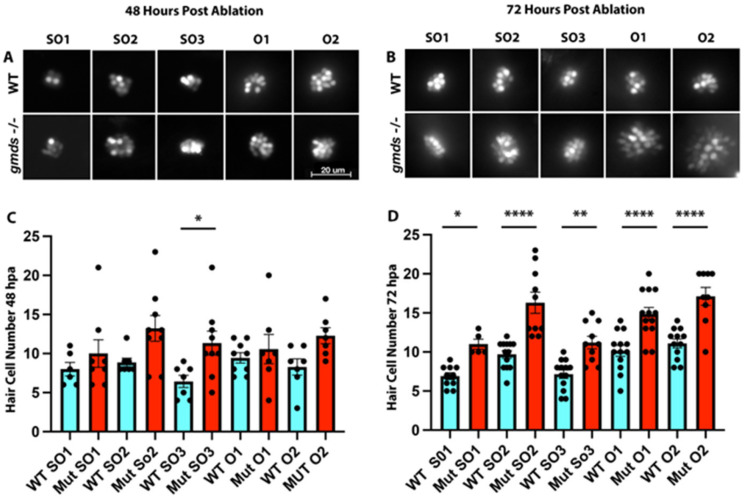
Quantitative assessment of increased hair cell regeneration of *gmds^−/−^* mutants with YO-PRO-1 staining. Data shown were from two independent experiments. At 48 hpa, a trend of increased hair cell number is observed in *gmds^−/−^* mutants, which is statistically significant for the SO3 neuromast (WT SO1 *n* = 6, Mut SO1 *n* = 8, WT SO2 *n* = 7, Mut SO2 *n* = 7, WT SO3 *n* = 7, Mut SO3 *n* = 9, WT O1 *n* = 9, Mut O1 *n* = 7, WT O2 *n* = 7, Mut O2 *n* = 6) (**A**,**C**). By 72 hpa, all assayed neuromasts show a statistically significant increase in hair cell number in *gmds^−/−^* mutants when compared to control siblings (WT SO1 *n* = 10, Mut SO1 *n* = 5, WT SO2 *n* = 12, Mut SO2 *n* = 13, WT SO3 *n* = 13, Mut SO3 *n* = 13, WT O1 *n* = 13, Mut O1 *n* = 13, WT O2 *n* = 12, Mut O2 *n* = 9) (**B**,**D**). Data presented as mean ± SEM, significance testing using a one-factor ANOVA with Sidak’s multiple comparisons test * *p* < 0.05, ** *p* < 0.01, **** *p* < 0.0001.

While the DASPEI and SEM-based experiments show faster regeneration of neuromast hair cells, it is unclear whether fully regenerated neuromast contain the correct number of hair cells. We set to quantify the number of hair cells in head neuromasts using YO-PRO-1 staining. Assessing the SO1, SO2, SO3, O1 and O2 neuromasts 48 h post-ablation, there is a trend of increased hair cell number in *gmds^−/−^* mutants; however, this was only statistically significant in the SO3 neuromast at 48 h post-ablation (Figure 7A,C). By 72 h post-ablation, when regeneration is complete, a statistically significant increase in hair cell number is observed in all tested neuromasts of the *gmds^−/−^* mutants when compared to wild-type siblings (Figure 7B,D).

### 2.7. Inhibition of Notch Signalling Affects Hair Cell Regeneration in gmds^−/−^ Mutants

**Figure 8:**
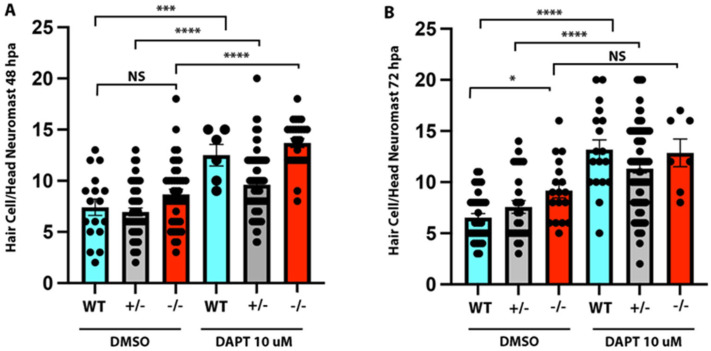
Inhibition of Notch signalling increases hair cell number after ablation in mutants and control groups (wild-type and *gmds +/-* siblings). Hair cell YO-PRO-1 counts 48 h post neomycin treatment, with DMSO control group (WT *n* = 17, +/− *n* = 27, −/− *n* = 31) or Notch inhibition with 10 µm DAPT group following ablation (WT *n* = 6, +/− *n* = 29, −/− *n* = 16) (**A**). Hair cell YO-PRO-1 counts 72 h post neomycin treatment, with DMSO control group (WT *n* = 18, +/− *n* = 20, −/− *n* = 17) or Notch inhibition with 10 µm DAPT treatment following ablation (WT *n* = 18, +/− *n* = 34, −/− *n* = 7) (**B**). Data presented as mean ± SEM, significance testing using a one-factor ANOVA with Sidak’s multiple comparisons test * *p* < 0.05, *** *p* < 0.001, **** *p* < 0.0001.

To determine the mechanism underlying increased neuromast and hair cell regeneration in the *gmds^−/−^* mutants, we examined the role of Notch signalling using the Notch signalling inhibitor DAPT. Notch receptor inhibition with DAPT at 10 µM significantly increased hair cell number in all genotypes at 48 hpa, while not reaching statistical significance in homozygous mutants at 72 hpa (*p* = 0.09) (Figure 8).

## 3. Discussion

Given that fucosylation of biomolecules by GDP-fucose plays an important role in organogenesis, development and regeneration, it is imperative to understand the effect of limited cellular GDP-fucose in animal model systems. Gmds regulates the rate-limiting step in the synthesis of GDP-fucose, which is observed in neuromast structures identified with lectin-based staining. While zebrafish *gmds^−/−^* swim in circles, often indicating defects with neuromast structure or subsequent neural transmission [6,7,26,27], *gmds^−/−^* neuromasts appear morphologically similar to their wild-type siblings. A small increase in hair cell number is observed under homeostatic conditions, which may contribute to this phenotype; however, defects in neurotransmission may also play a role, as observed in other *gmds* mutant strains [34]. The previously published zebrafish *gmds* (*gmds^sly^*) mutants also have a swimming phenotype with increased tail C-bends but did not swim in circles [28,34]. This phenotype was attributed to increased number and size of neuromuscular synapses. While we observed this increased neuromuscular junction size in some *gmds^−/−^* (*gmds^nfl2^*) embryos, there is considerable variability, with some embryos showing decreased synapse number and size (Supplemental Figure S1). Therefore, it is possible that reduced hair cell number and synaptic density, both increased and decreased, contribute to the circular swimming phenotype in *gmds^−/−^* mutants.

Given the high level of *gmds* expression in zebrafish neuromast support cells [20], and its known role in regulating Notch signalling (which must be downregulated at the earliest stages of regeneration) [5,10,16,17], we hypothesized that *gmds* homozygous mutants would have increased neuromast hair cell regenerative capacity. The lack of regenerative capacity of mammalian hair cells in the inner ear can lead to hearing loss, and thus the understanding of regenerative hair cell mechanisms in lower animals such as zebrafish could provide valuable insights into genetic targets for therapeutic intervention.

Our data has also provided insight to the timeline of hair cell structural re-appearance after initially being destroyed due to neomycin treatment. Zebrafish normally regenerate hair cells within 72 h after damage [35], and this is confirmed in control siblings using both DASPEI and YO-PRO-1 staining, along with SEM imaging. In *gmds^−/−^* mutants, complete regeneration is faster, with metabolically active hair cells that display both kinocilia and stereocilia by 48 h post-ablation. By 72 h post-ablation, an increase in the number of hair cells is observed in *gmds^−/−^* neuromasts, implying increased proliferation of support cells; however, this was not directly tested as part of this study. These data demonstrate that downregulated fucosylation promotes hair cell regeneration. Both trunk and head neuromasts displayed faster regeneration, however, the analysis of hair cell number was quantified in head neuromasts only, thus it cannot be said for certain that trunk neuromasts also contain increased numbers of hair cells after regeneration.

The quantification of regenerated neuromast hair cell clusters in the *gmds^−/−^* mutants revealed the robustness of zebrafish in regenerating damaged or functionally dead neuromast hair cells after ototoxic damage. DASPEI and YO-PRO-1 dyes are internalized by live hair cells through mechanotransduction channels [36], thus showing that the regenerated hair cells contain active mechanotransductive channels required for coordinating movement. This, along with SEM data showing the presence of stereocilia and kinocilia in the regenerated neuromast hair cell clusters, implies regenerated neuromasts are functional, although behavioural or electrophysiological assays to check the functionality were not part of this study.

Notch, Wnt and Fgf signalling pathways have been reported to play key roles in the regeneration of damaged hair cells in zebrafish and other animal hair cells [5,10,35]. Similar phenotypes were observed between *fgf3^−/−^* and *gmds^−/−^* mutants. *fgf3^−/−^* mutants, or pan- FGF inhibition with chemical inhibitors, caused faster and supernumerary hair cell regeneration after neomycin ablation [10]. This was attributed to increased Wnt-dependent support cell proliferation after ablation. RNA sequencing of neuromast support cells showed a downregulation of *gmds* and *fut8* (a fucosyltransferase gene) in *fgf3^−/−^* mutants, which could contribute to the observed regenerative phenotype [10]. Current models indicate that FGF and Notch inhibit Wnt-dependent proliferation through parallel pathways; however, the identification of reduced *gmds* expression in *fgf3^−/−^* mutants highlights the possibility that Fgf regulates Notch signalling through Gmds, given the known role of the fucosylation of Notch receptors’ Egf-like repeats required for ligand binding [37].

Current models demonstrate that FGF and Notch signalling operate in parallel to modify Wnt-dependent proliferation of support cells in neuromasts after hair cell ablation. Our DAPT treatment data shows that Notch inhibition further increases hair cell number and regenerative capacity in *gmds^−/−^* mutants, heterozygotes and wild-type siblings. However, the increase in hair cell number after regeneration did not reach statistical significance in *gmds* homozygotes at 72 hpa, implying that Notch signalling may be partially affected in these larvae. We have not found any reports of Fgf receptors requiring fucosylation, and *gmds* likely functions downstream of FGF signalling, given its downregulation in *fgf3* mutant hair cells [10]. A direct effect on Wnt signalling is possible given that the fucosylation of the canonical Wnt co-receptor LPR6 reduces Wnt/B-Catenin signalling [38], and thus a loss of *gmds* could positively affect Wnt-dependent proliferation in neuromast support cells; however, this was not tested as part of this study.

Other pathways could be implicated in this regenerative phenotype, such as the TGF-Beta pathway that is altered in the super regenerating *mgat5a^−/−^* zebrafish mutants [39]. Interestingly, this gene encodes a beta1,6-*N*-acetylglucosaminyl-transferase enzyme active in the Golgi, underscoring the importance of glycosylation for hair cell regeneration. TGF-Beta receptors 1 and 2 have been reported to require core fucosylation by FUT8 for optimal function [40,41], indicating that TGF-Beta signalling may also play a role in hair cell regeneration in a fucosylation dependent manner. Also, Notch and TGF-Beta intracellular transducers (NICD and Smad3) have been shown to form functional synergistic interactions to regulate transcription of Notch genes [42]. It is thus possible that loss *gmds* may affect TGF-β signalling, whether through reduced fucosylation of TGF-β receptors or through interactions between Smad3 and NICD; however, this was not tested as part of this study.

Our data clearly shows that mutation of *gmds* in zebrafish increased the regenerative capacity of neuromast hair cells after neomycin damage. In contrast, it has been previously shown that supplementation of zebrafish with fucoidan, a naturally occurring seaweed containing high levels of fucose, improved hair cell regeneration after damage by neomycin [43]. Fucoidan also contains galactose, mannose, rhamnose, glucose and glucuronic acid, which could account for these differences. Also, while supplementation with fucoidan would presumably lead to increased fucosylation via the salvage pathway, levels of fucosylation were not tested and thus the increase in regenerative capacity with fucoidan supplementation cannot be directly attributed to changes in fucosylation.

It is important to note that the CRISPR-induced mutation of *gmds* has a lethal effect, with *gmds^−/−^* mutants dying before 10 days post-fertilization. This underscores the complications of using GMDS inhibition for hearing loss therapeutic development and its resultant effect on organismal health. *GMDS* temporary or localized inhibition could potentially be therapeutically beneficial to promote hair cell regeneration. For example, the use of cell-permeable fucose mimics that compete for fucosyltransferase binding [44,45], currently being developed for anti-cancer treatments, could represent a possible therapeutic avenue for regeneration of human hair cells if delivered locally to the inner ear.

## 4. Materials and Methods

### 4.1. Zebrafish Husbandry

We previously generated a zebrafish *gmds* CRISPR loss of function mutant line (*gmds^nfl2^*) [20]. Homozygous mutant larvae were generated from heterozygous in-crosses, with heterozygotes and wild-type siblings used as controls. All fish were reared and managed with standards set by the Memorial University of Newfoundland’s Animal Care Committee and the Canadian Council on Animal Care. To enable clear imaging of neuromast hair cells with vital dyes, embryos were treated with 0.003% 1-phenyl 2-thiourea (PTU: Millipore Sigma, St. Louis, MO, USA) after 24 h post-fertilization to prevent zebrafish pigment formation. For most experiments, homozygous *gmds* mutant embryos were identified with either cerebral hemorrhage and/or curly tail. For Notch inhibition experiments, all larvae were genotyped using Sanger sequencing.

### 4.2. In Situ Hybridization Gene Expression

In situ hybridization experiments used for positional expression of selected genes in whole mounted larvae were performed using previously published protocols [46]. Briefly, in situ hybridization probes were generated from total RNA pool extracted from zebrafish embryos. One-Step Superscript IV RT-PCR kits (Invitrogen, Carlsbad, CA, USA) were used to synthesize PCR amplicons that were later used for the specific gene RNA probe production. An incorporated T7 RNA promoter was used to produce an antisense probe, labelled with DIG (Roche, Basal, Switzerland). In situ hybridization experiments require fixing embryos in 4% paraformaldehyde (PFA: Millipore Sigma, St. Louis, MO, USA), with post-fixation permeabilization using Proteinase K. In situ hybridization experiments for *gmds*, *slc17a8*, *fut9b* and *sox2* genes were performed at 72 hpf. Images were taken with a Nikon Brightfield Stereoscope (Nikon SMZ18, Tokyo, Japan).

### 4.3. AAL Staining for Fucose Detection and Immunohistochemistry

*Aleuria aurantia* Lectin (AAL, Vector Labs, Burlingame, CA, USA) stain was used to detect fucosylated proteins in the WT and *gmds* mutant embryos at 72 hpf. Live embryos were placed in centrifuge tubes containing 1 mL of 20 µg/mL AAL for 10 min and washed twice in PBST for 5 min, with larvae imaged on a Nikon SMZ18 microscope with a wide-pass green filter.

For immunohistochemistry, anti-Hnk-1 (1/250, Zirc, Eugene, OR, USA) and anti-acetylated tubulin primary antibodies (1/1000, Millipore Sigma, St. Louis, MO, USA) were used. 4% paraformaldehyde was used to fix embryos for anti-Hnk-1 immunohistochemistry, and Dent’s fixative (80%methanol/20% DMSO) was used for anti-acetylated tubulin immunohistochemistry. Embryos were blocked in 5% goat serum with 2% BSA before primary antibody treatment. For antibody visualization, we utilized goat anti-mouse 568 Alexa-Fluor secondary antibody (Thermofisher, Waltham, MA, USA), imaged using a Zeiss Airyscan confocal microscope (Oberkochen, Germany).

### 4.4. Hair Cell Damage Induction

Using a modified version of previously published neomycin ablation procedure [47], zebrafish larvae at five days post-fertilization (dpf) were exposed to 500 µM neomycin (Neomycin sulphate; Millipore Sigma, St. Louis, MO, USA) for 1 h to ablate neuromast hair cells. The larvae were exposed to the neomycin solution for 60 min at 28 °C in standard petri dishes, allowing for uniform diffusion and effective chemical injury across neuromasts. After the final rinse, larvae were transferred into clean embryo medium at 28 °C for recovery prior to further processing for imaging assays with vital dyes or for scanning electron microscopy (SEM) imaging.

### 4.5. Neuromast Regeneration Time-Lapse Imaging with DASPEI

To assess the rate of neuromast regeneration following neomycin-induced hair cell damage, embryos were stained with 2-(4-(dimethylamino)styryl)-N-ethylpyridinium iodide (DASPEI; Millipore Sigma, St. Louis, MO, USA), a vital dye that selectively labels metabolically active hair cells within a neuromast by accumulation in the mitochondria of live cells [47,48]. A total of 0.05% DASPEI solution was freshly prepared in PTU-embryo medium. At time points (4, 24, 48, and 72 h) post-neomycin treatment, zebrafish larvae were gently rinsed and then incubated in the DASPEI solution for 20 min at 28 °C in the dark to prevent photobleaching. After staining, larvae were washed three times with PTU-containing embryo medium. For imaging, embryos were briefly anesthetized in 10 µg/mL tricaine solution prepared in PTU medium. Embryos were mounted in a drop of 6% methylcellulose and visualized using a Nikon SMZ18 fluorescence stereomicroscope equipped with a 488 nm green filter to detect DASPEI-positive cells.

### 4.6. YO-PRO-1 Hair Cell Quantification Under Homeostatic Conditions and After Ablation

To determine the number of hair cells in neuromasts under homeostatic conditions, zebrafish embryos were stained with YO-PRO-1 iodide (Invitrogen, Eugene, OR, USA). This dye selectively labels mechanosensory hair cells and allows for their visualization and quantification in vivo [36]. Larvae were stained using a 3 μM working solution of YO-PRO-1, freshly prepared in PTU embryo media. Fish were placed in this solution and incubated at 28 °C for 20 min in the dark. After staining, fish were washed in clean PTU media. For imaging, embryos were anesthetized in 0.02% tricaine (Ethyl 3-aminobenzoate methanesulfonate (Millipore Sigma, St. Louis, MO, USA) prepared in PTU medium for imaging. Fluorescence imaging was performed using a Nikon (Tokyo, Japan) Eclipse Ti2 microscope equipped with a green filter set suitable for YO-PRO-1 (green) emission at 509 nm. Imaging and quantification were focused on head neuromasts. The YO-PRO-1 staining procedure was also used after ablation with neomycin, as previously described, to quantify hair cell number at 48 and 72 h post-ablation.

### 4.7. Scanning Electron Microscopy (SEM) of Hair Cell Regeneration

To visualize structural changes during neuromast hair cell regeneration, SEM imaging was performed during homeostasis on 5 dpf larvae, and on neomycin-treated larvae (treated at 5 dpf, analyzed at 1, 2, and 3 days post-ablation). Whole larvae were fixed overnight at 4 °C in a solution containing 2.5% glutaraldehyde and 2.5% paraformaldehyde (Electron Microscopy Sciences, Hatfield, PA, USA) in 0.1 M Sodium Cacodylate buffer (Electron Microscopy Sciences, Hatfield, PA, USA). Following fixation, the embryos were washed three times for 10 min each in fresh Sodium Cacodylate buffer. Embryos were then placed in 1% Sodium Tetroxide (OSO_4_) in 0.1 M Sodium Cacodylate buffer for 1 h at room temperature and again washed twice in a new Sodium Cacodylate buffer. Embryos were then dehydrated through a graded ethanol series and then washed into a graded ethanol/hexamethyldisilazane (HMDS, Electron Microscopy Sciences, Hatfield, PA, USA) series, and then into 100% HMDS for overnight drying. Samples were gold plated and imaged at 12,230× on a SEM (FEI Quanta 400, Hillsboro, Oregon, USA) in partial vacuum mode.

### 4.8. Inhibition of Notch Signalling During Regeneration

To investigate the molecular mechanism regulating the speed and capacity of hair cell regeneration in *gmds^−/−^* mutants, we targeted Notch signalling with DAPT((2S)–N-[(3,5-Difluorophenyl)acetyl]-L-alanyl-2-phenyl]glycine 1,1-dimethylethyl ester), as previously reported [20]. A group of ablated embryos were incubated either in PTU-embryo-containing DAPT at 10 µM final concentration or in DMSO (vehicle control). Whole embryos from each group were incubated with YO-PRO-1 and imaged for head neuromast hair cells at 48 h and 72 h post-ablation using a Nikon Ti2 Eclipse fluorescent microscope. Hair cells were counted with the researcher masked to genotype (counting was performed before genotyping). Larvae were genotyped with sanger sequencing after YO-PRO-1 imaging and hair cell counting.

### 4.9. Data Preparation and Statistical Analysis

All experiments were repeated 2–4×, with specific (*n*) values stated in figure legends. One-factor ANOVAs were used for statistical significance when noted in figure legends. Artificial Intelligence was not used to generate text for Figures for this document.

## 5. Conclusions

In summary, we have shown that mutation of the *gmds* gene, important for making GDP-fucose, causes increased hair cell number in zebrafish neuromasts and a further increase in hair cell number during post-ablation regeneration. A faster rate of regeneration is also observed. These findings are significant because zebrafish neuromast hair cells closely resemble hair cells in the human inner ear, which cannot regenerate after damage, causing permanent hearing loss. The increased regeneration observed in the *gmds^−/−^* mutants offers a useful model to understand the biological processes behind hair cell regeneration after damage by ageing, ototoxic medication, noise or due to genetic mutation. Targeting GDP-fucose cellular availability in a localized or conditional manner represents a possible approach for therapeutic benefit to induce regeneration in mammalian or human hair cells.

## Data Availability

All data can be obtained from the corresponding author upon request.

## Supplementary Materials

The following supporting information can be downloaded at: https://www.mdpi.com/article/10.3390/ijms26199737/s1.
