# Supplementary material for: Mutation of the GDP-Fucose Biosynthesis Gene gmds Increases Hair Cell Number and Neuromast Regenerative Capacity in Zebrafish
Source: Int J Mol Sci. 2025 Oct 7;26(19):9737. doi: 10.3390/ijms26199737 (PMC12524676; doi:10.3390/ijms26199737)
Supplement: Supplementary file 1 [file ijms-26-09737-s001.zip › ijms-3825091-supplementary/Supplementary files/IJMS- Supplemental Data word file.pdf]

Supplemental Data

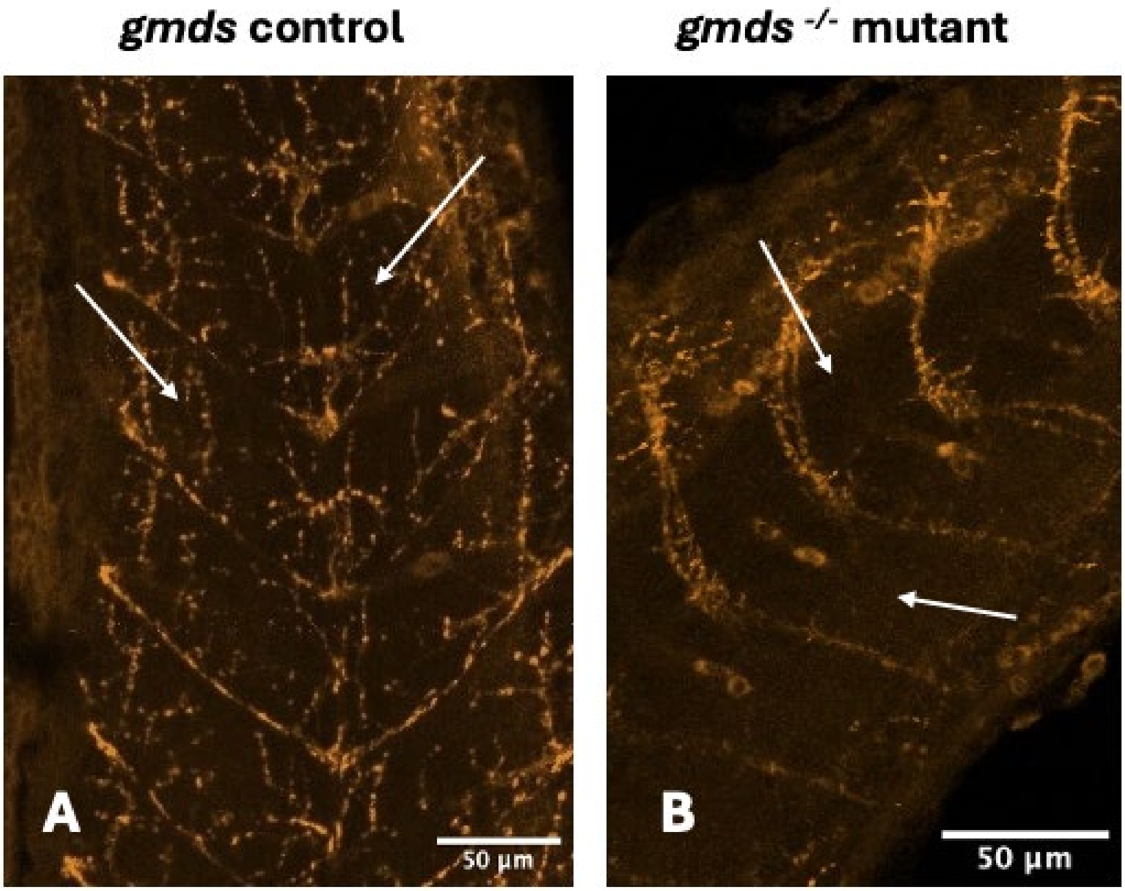

Supplemental Figure S1: Immunohistochemistry (IHC) staining for synaptic vesicles membrane protein, SV2. 4 out of 5 *gmds*$^{-/-}$ have reduced synaptic vesicle content between motor neurons. (A) shows intact synaptic vesicle protein density at 48 hpf in wild type siblings. (B) shows missing synaptic vesicle protein density in the *gmds-/-* mutants group at 48 hpf. The white arrows show the area of missing SV-2 protein in the trunk tail of the larvae. Experiment was performed twice with 5 different larvae for each group of control and *gmds-/-* mutants. Z-stack images were taken at 200X and presented as maximum projection with Fiji/ImageJ Z-project package.
